# The prognostic value of artificial intelligence to predict cardiac amyloidosis in patients with severe aortic stenosis undergoing transcatheter aortic valve replacement

**DOI:** 10.1093/ehjdh/ztae022

**Published:** 2024-03-13

**Authors:** Milagros Pereyra Pietri, Juan M Farina, Ahmed K Mahmoud, Isabel G Scalia, Francesca Galasso, Michael E Killian, Mustafa Suppah, Courtney R Kenyon, Laura M Koepke, Ratnasari Padang, Chieh-Ju Chao, John P Sweeney, F David Fortuin, Mackram F Eleid, Kristen A Sell-Dottin, David E Steidley, Luis R Scott, Rafael Fonseca, Francisco Lopez-Jimenez, Zachi I Attia, Angela Dispenzieri, Martha Grogan, Julie L Rosenthal, Reza Arsanjani, Chadi Ayoub

**Affiliations:** Department of Cardiovascular Medicine, Mayo Clinic, 5777 East Mayo Boulevard, Phoenix, AZ 85054, USA; Department of Cardiovascular Medicine, Mayo Clinic, 5777 East Mayo Boulevard, Phoenix, AZ 85054, USA; Department of Cardiovascular Medicine, Mayo Clinic, 5777 East Mayo Boulevard, Phoenix, AZ 85054, USA; Department of Cardiovascular Medicine, Mayo Clinic, 5777 East Mayo Boulevard, Phoenix, AZ 85054, USA; Department of Cardiovascular Medicine, Mayo Clinic, 5777 East Mayo Boulevard, Phoenix, AZ 85054, USA; Department of Cardiovascular Medicine, Mayo Clinic, 5777 East Mayo Boulevard, Phoenix, AZ 85054, USA; Department of Cardiovascular Medicine, Mayo Clinic, 5777 East Mayo Boulevard, Phoenix, AZ 85054, USA; Department of Cardiovascular Medicine, Mayo Clinic, 5777 East Mayo Boulevard, Phoenix, AZ 85054, USA; Department of Cardiovascular Medicine, Mayo Clinic, 5777 East Mayo Boulevard, Phoenix, AZ 85054, USA; Department of Cardiovascular Medicine, Mayo Clinic, Rochester, MN, USA; Department of Cardiovascular Medicine, Mayo Clinic, Rochester, MN, USA; Department of Cardiovascular Medicine, Mayo Clinic, 5777 East Mayo Boulevard, Phoenix, AZ 85054, USA; Department of Cardiovascular Medicine, Mayo Clinic, 5777 East Mayo Boulevard, Phoenix, AZ 85054, USA; Department of Cardiovascular Medicine, Mayo Clinic, Rochester, MN, USA; Department of Cardiovascular Surgery, Mayo Clinic, Phoenix, AZ, USA; Department of Cardiovascular Medicine, Mayo Clinic, 5777 East Mayo Boulevard, Phoenix, AZ 85054, USA; Department of Cardiovascular Medicine, Mayo Clinic, 5777 East Mayo Boulevard, Phoenix, AZ 85054, USA; Department of Cardiovascular Medicine, Mayo Clinic, 5777 East Mayo Boulevard, Phoenix, AZ 85054, USA; Department of Cardiovascular Medicine, Mayo Clinic, Rochester, MN, USA; Department of Cardiovascular Medicine, Mayo Clinic, Rochester, MN, USA; Department of Hematology, Mayo Clinic, Rochester, MN, USA; Department of Cardiovascular Medicine, Mayo Clinic, Rochester, MN, USA; Department of Cardiovascular Medicine, Mayo Clinic, 5777 East Mayo Boulevard, Phoenix, AZ 85054, USA; Department of Cardiovascular Medicine, Mayo Clinic, 5777 East Mayo Boulevard, Phoenix, AZ 85054, USA; Department of Cardiovascular Medicine, Mayo Clinic, 5777 East Mayo Boulevard, Phoenix, AZ 85054, USA

**Keywords:** Transcatheter aortic valve replacement, Cardiac amyloidosis, Artificial intelligence

## Abstract

**Aims:**

Cardiac amyloidosis (CA) is common in patients with severe aortic stenosis (AS) undergoing transcatheter aortic valve replacement (TAVR). Cardiac amyloidosis has poor outcomes, and its assessment in all TAVR patients is costly and challenging. Electrocardiogram (ECG) artificial intelligence (AI) algorithms that screen for CA may be useful to identify at-risk patients.

**Methods and results:**

In this retrospective analysis of our institutional National Cardiovascular Disease Registry (NCDR)-TAVR database, patients undergoing TAVR between January 2012 and December 2018 were included. Pre-TAVR CA probability was analysed by an ECG AI predictive model, with >50% risk defined as high probability for CA. Univariable and propensity score covariate adjustment analyses using Cox regression were performed to compare clinical outcomes between patients with high CA probability vs. those with low probability at 1-year follow-up after TAVR. Of 1426 patients who underwent TAVR (mean age 81.0 ± 8.5 years, 57.6% male), 349 (24.4%) had high CA probability on pre-procedure ECG. Only 17 (1.2%) had a clinical diagnosis of CA. After multivariable adjustment, high probability of CA by ECG AI algorithm was significantly associated with increased all-cause mortality [hazard ratio (HR) 1.40, 95% confidence interval (CI) 1.01–1.96, *P* = 0.046] and higher rates of major adverse cardiovascular events (transient ischaemic attack (TIA)/stroke, myocardial infarction, and heart failure hospitalizations] (HR 1.36, 95% CI 1.01–1.82, *P* = 0.041), driven primarily by heart failure hospitalizations (HR 1.58, 95% CI 1.13–2.20, *P* = 0.008) at 1-year follow-up. There were no significant differences in TIA/stroke or myocardial infarction.

**Conclusion:**

Artificial intelligence applied to pre-TAVR ECGs identifies a subgroup at higher risk of clinical events. These targeted patients may benefit from further diagnostic evaluation for CA.

## Graphical Abstract

**Graphical Abstract:**
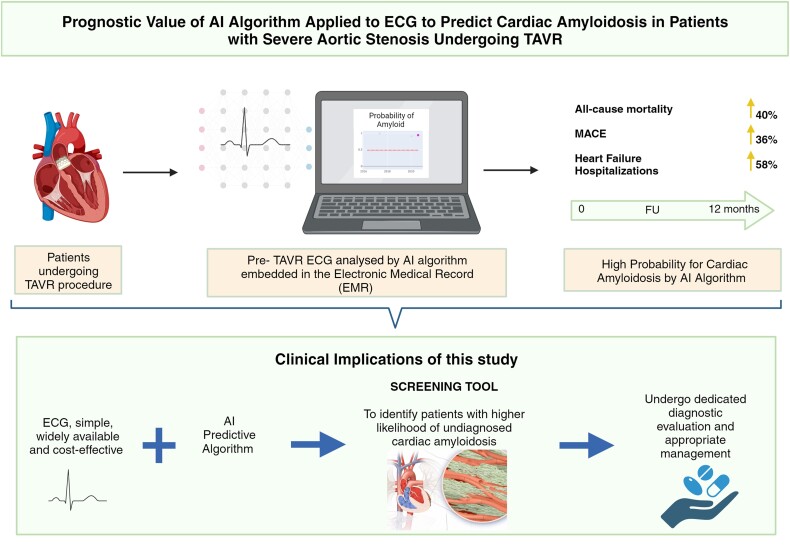

## Introduction

Transcatheter aortic valve replacement (TAVR) is increasingly and widely utilized to treat severe aortic stenosis (AS).^1,2^ Cardiac amyloidosis (CA), an infiltrative disease caused by the deposition of abnormal protein (amyloid) in cardiac tissue, is associated with poor prognosis.^3,4^ Transthyretin (TTR) CA increases in prevalence with age and has been observed to be more common in patients with AS undergoing TAVR, with several studies showing up to 16% prevalence when comprehensive workup is undertaken.^5,6^ However, assessment for TTR CA in all TAVR patients is practically challenging and costly and not routinely undertaken.

Artificial intelligence (AI) applications for screening and detection of pathology in the clinical setting are rapidly expanding.^7,8^ Artificial intelligence applied to electrocardiogram (ECG) has been validated for the screening and detection of CA.^9^ We, therefore, sought to evaluate the prognostic value and utility of the previously validated ECG AI algorithm to assess for increased probability of CA in patients with severe AS undergoing TAVR. We hypothesized that patients identified by the clinically available AI algorithm applied to pre-TAVR ECG as increased probability for CA may have worse clinical outcomes and that AI may be clinically useful to screen and identify higher-risk patients who may then be better selected to undergo targeted clinical evaluation for CA.

## Methods

### Study population

The retrospective study was approved by the Mayo Clinic Institutional Review Board (IRB). All included patients provided consent for enrolment into the database, and consent was waived for retrospective review of the data by the IRB. Adult (age > 18 years) patients who underwent TAVR between January 2012 and December 2018 at the three Mayo Clinic sites (Rochester, MN; Phoenix, AZ; and Jacksonville, FL) and had a 12-lead ECG within 1 month prior to TAVR were identified from the Mayo Clinic institutional NCDR-TAVR database. If more than one baseline ECG was available from this specified timeframe, the closest ECG to the TAVR procedure date was selected for analysis.

### Electrocardiogram artificial intelligence algorithm

**Figure 1:**
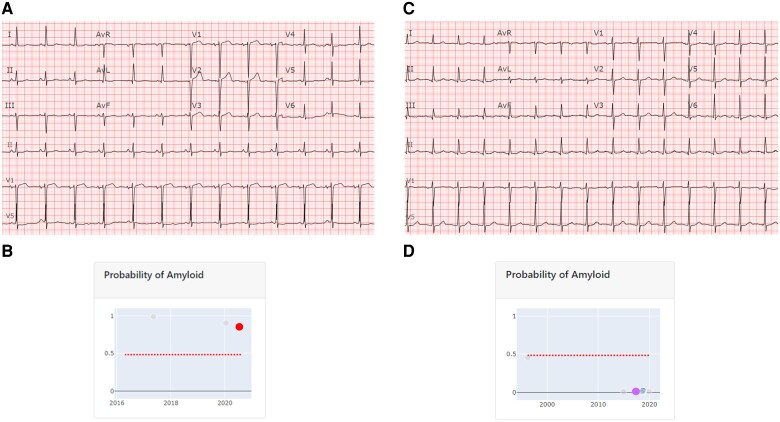
Artificial intelligence applied to electrocardiograms. *Panel A* demonstrates 12-lead electrocardiogram of a patient flagged as high probability for cardiac amyloidosis by the artificial intelligence algorithm as demonstrated in *Panel 1B*, with probability for cardiac amyloidosis predicted at 98.9% (red dot). In the contrast, with normal electrocardiogram (*C*) and low probability for cardiac amyloidosis at 0.03% predicted by the electrocardiogram artificial intelligence (purple dot) in *D*.

Pre-TAVR ECGs were retrieved and analysed by an AI predictive algorithm embedded in the institutional electronic medical record (EMR), from which predictive risk scores were collected for CA probability (*Figure 1*). High CA probability was defined as >50% risk prediction by the ECG AI algorithm predictive model, as noted in previously published work validating the diagnostic value of this model for CA.^9,10^ Percentage predicted probability for CA for each ECG was recorded, and the cohort grouped into those with high risk for CA vs. those at low risk. Additionally, comparison was made of patients per quartile of ECG AI probability for CA.

### Clinical data

Baseline demographics, cardiovascular risk factors and comorbidities, echocardiographic data including left ventricular (LV) ejection fraction, right ventricular systolic pressure, and LV wall thickness, and laboratory values were extracted from the NCDR-TAVR database. Electronic medical records were chart reviewed to assess for a clinical diagnosis of CA, Technetium pyrophosphate (PYP) scan, free light chain (FLC) assay, and endomyocardial biopsy. Clinical outcomes including all-cause mortality, heart failure (HF) hospitalizations, transient ischaemic attack (TIA)/stroke, and myocardial infarction (MI) at 1-year follow-up after the TAVR procedure were extracted from the NCDR-TAVR database. A composite of major adverse cardiovascular events (MACE), defined as HF hospitalizations, MI, and ischaemic stroke was also evaluated.

### Statistical analysis

Continuous variables were summarized as mean and standard deviation (mean ± SD) or median and interquartile range according to distribution, while categorical variables were presented as frequencies with percentages. Independent samples *t*-test or non-parametric tests were used to compare continuous variables and chi-square to compare categorical variables.

The association between the risk of CA as predicted by the ECG AI tool and clinical outcomes at 1-year follow-up was summarized with hazard ratios (HRs) and 95% confidence intervals (CIs), estimated with Cox regression models. Univariable and propensity score covariate adjustment analyses were performed. For the propensity score covariate adjustment, a propensity score was built based on demographics and comorbidities (age, sex, hypertension, prior stroke/TIA, prior MI, diabetes, smoking, prior HF history, history of atrial fibrillation, conduction abnormalities, prior pacemaker or defibrillator, and prior coronary revascularization), and covariate adjustment using the propensity score was applied.^11^ The proportional hazard assumption was tested by plotting the log–minus–log survival function against the log(time). Kaplan–Meier curves were used to show survival between the two groups. *P*-values of <0.05 were considered statistically significant for all analyses. Statistical analyses were conducted using IBM SPSS Statistics software, version 28.0 (IBM SPSS Inc., Armonk, NY, USA).

## Results

A total of 1426 patients were included, with mean age 81.0 ± 8.5 years and 57.6% were male. A review of EMR identified clinical diagnosis of CA in only 17 patients (1.2%) undergoing TAVR. Pyrophosphate scans were available in 25 patients (1.7%) from the cohort, with 7 (0.5%) being positive for TTR CA. Serum FLC was measured in 170 patients (11.9%) with a kappa/lambda ratio > 3 present in 17 (1.2%). A high probability of CA was identified in 349 patients (24.4%) by the ECG AI algorithm. Patients with high probability for CA were more likely to be male (69.9% vs. 53.6%, *P* < 0.001), to have a history of hypertension (88.2% vs. 82.3%, *P* = 0.009), diabetes (38.9% vs. 32.0%, *P* = 0.017) HF (81.3% vs. 75.9%, *P* = 0.035), stroke/TIA (19.7% vs. 14.5%, *P* = 0.021), MI (29.7% vs. 22.7%, *P* = 0.007), revascularization (45.5% vs. 35.4%, *P* < 0.001), and pacemaker or defibrillator implantation (27.7% vs. 14.2%, *P* < 0.001) (*Table 1*). A total of 144 patients (41.2%) of the patients identified as high risk for CA were on anticoagulation, predominantly for atrial fibrillation.

**Table 1:** Baseline characteristics

	Overall (*n* = 1426)	Low amyloid probability (*n* = 1077)	High amyloid probability (*n* = 349)	*P*-value
Age, years	81.0 ± 8.5	81.2 ± 8.1	80.5 ± 9.4	0.205
**Sex, *n* (%)**				
Male	821 (57.6%)	577 (53.6%)	244 (69.9%)	
Female	605 (42.4%)	500 (46.4%)	105 (30.1%)	<0.001
**Comorbidities, *n* (%)**				
Hypertension	1195 (83.8%)	887 (82.3%)	308 (88.2%)	0.009
Prior stroke/TIA	226 (15.8%)	157 (14.5%)	69 (19.7%)	0.021
Prior myocardial infarction/Coronary artery disease	349 (24.4%)	245 (22.7%)	104 (29.7%)	0.007
Diabetes mellitus	481 (33.7%)	345 (32.0%)	136 (38.9%)	0.017
Smoking	191 (13.3%)	135 (12.5%)	56 (16.0%)	0.094
Heart failure	1102 (77.2%)	818 (75.9%)	284 (81.3%)	0.035
History of atrial fibrillation/atrial flutter	608 (42.6%)	427 (39.6%)	181 (51.8%)	<0.001
Conduction abnormalities				
Left bundle branch block	148 (10.3%)	94 (8.7%)	54 (15.4%)	<0.001
First degree atrioventricular block	298 (20.8%)	229 (21.2%)	69 (19.7%)	0.551
Second degree AV block	42 (2.9%)	23 (2.1%)	19 (5.4%)	0.001
Prior pacemaker or defibrillator	251 (17.6%)	154 (14.2%)	97 (27.7%)	<0.001
Prior revascularization^a^	541 (37.9%)	382 (35.4%)	159 (45.5%)	<0.001
**NYHA (New York Heart Association) class, *n*(%)**				
Class I	119 (8.3%)	94 (8.7%)	25 (7.1%)	
Class II	368 (25.8%)	283 (26.2%)	85 (24.3%)	
Class III	768 (53.8%)	577 (53.5%)	191 (54.7%)	
Class IV	171 (11.9%)	123 (11.4%)	48 (13.7%)	0.491
**Laboratory values**				
Haemoglobin, g/dL	12.1 ± 1.9	12.1 ± 1.8	12.1 ± 2.1	0.828
Creatinine, mg/dL	1.3 ± 1.0	1.3 ± 1.0	1.5 ± 1.2	0.002
Albumin, g/dL	4.1 ± 0.3	4.1 ± 0.3	4.1 ± 0.4	0.429
**Transthoracic echocardiogram**				
Ejection fraction, %	57.4 ± 12.9	58.9 ± 12.0	53.0 ± 14.5	<0.001
Right ventricular systolic pressure, mmHg	41.6 ± 14.1	40.6 ± 13.6	44.6 ± 15.1	<0.001
Interventricular septum thickness, mm	12.4 ± 2.5	12.3 ± 2.1	12.4 ± 3.2	0.657
Left ventricular posterior wall thickness, mm	11.5 ± 2.1	11.5 ± 1.9	11.5 ± 2.6	0.777

^a^Prior coronary artery bypass grafting and percutaneous coronary intervention.

A total of 171 patients (11.9% of the study population) died during 1-year follow-up. Fifty-five deaths (15.7%) occurred in the high probability for CA group and 116 (10.7%) in the low probability group (unadjusted HR 1.51, 95% CI 1.10–2.08, *P* = 0.012). In total, 221 patients (15.5%) experienced a MACE during 1-year follow-up, from which 70 (20.0%) occurred in the high probability for CA group vs. 151 (14.0%) in the low probability group (unadjusted HR 1.48, 95% CI 1.12–1.97, *P* = 0.006). Among MACE, HF hospitalizations were the most frequent events, with an incidence of 16.3% in the high CA probability group and 9.9% in the low probability group (unadjusted HR 1.69, 95% CI 1.23–2.33, *P* = 0.001).

**Figure 2:**
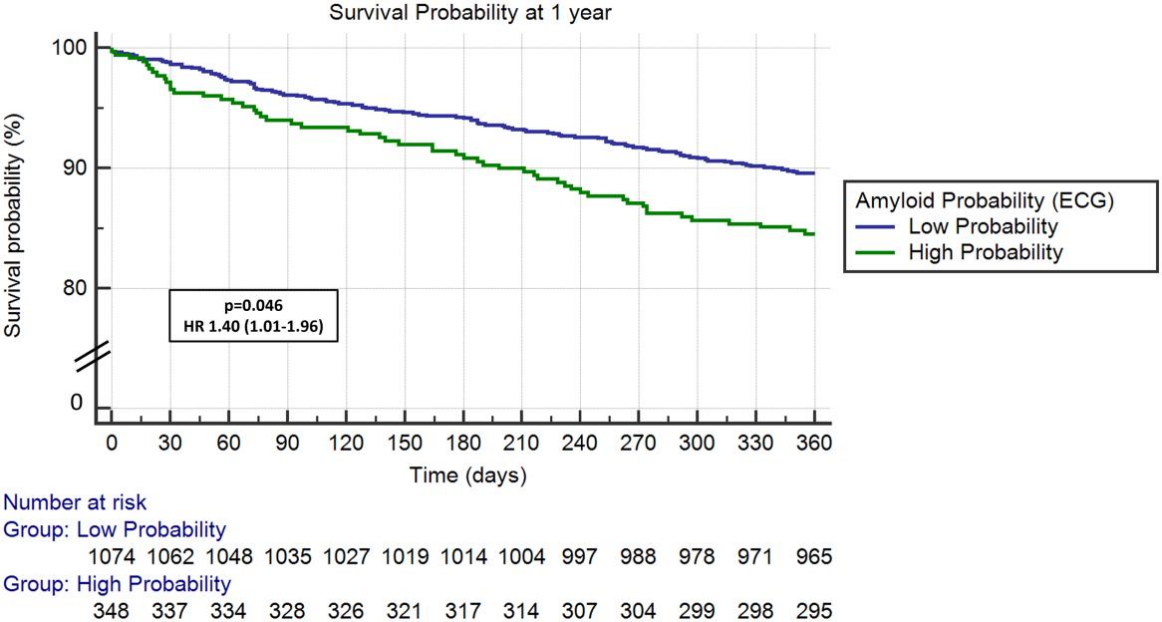
All-cause mortality in transcatheter aortic valve replacement patients with high vs. low probability for cardiac amyloidosis. ECG, electrocardiogram; HR, hazard ratio.

**Figure 3:**
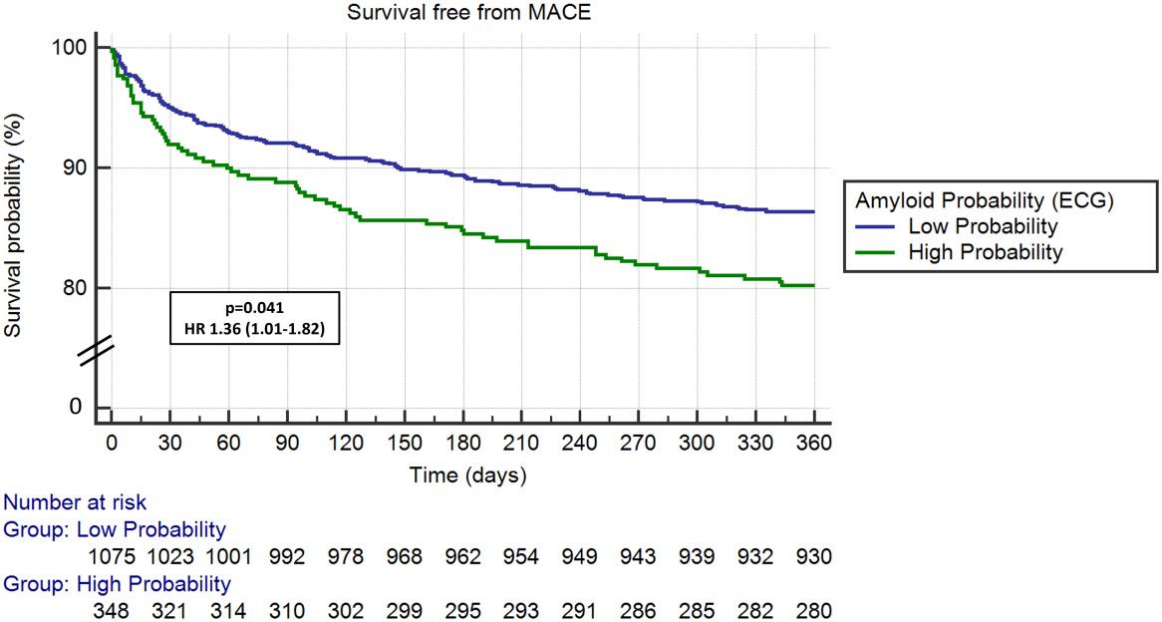
Major adverse cardiovascular events in transcatheter aortic valve replacement patients with high vs. low probability for cardiac amyloidosis. ECG, electrocardiogram; HR, hazard ratio.

**Figure 4:**
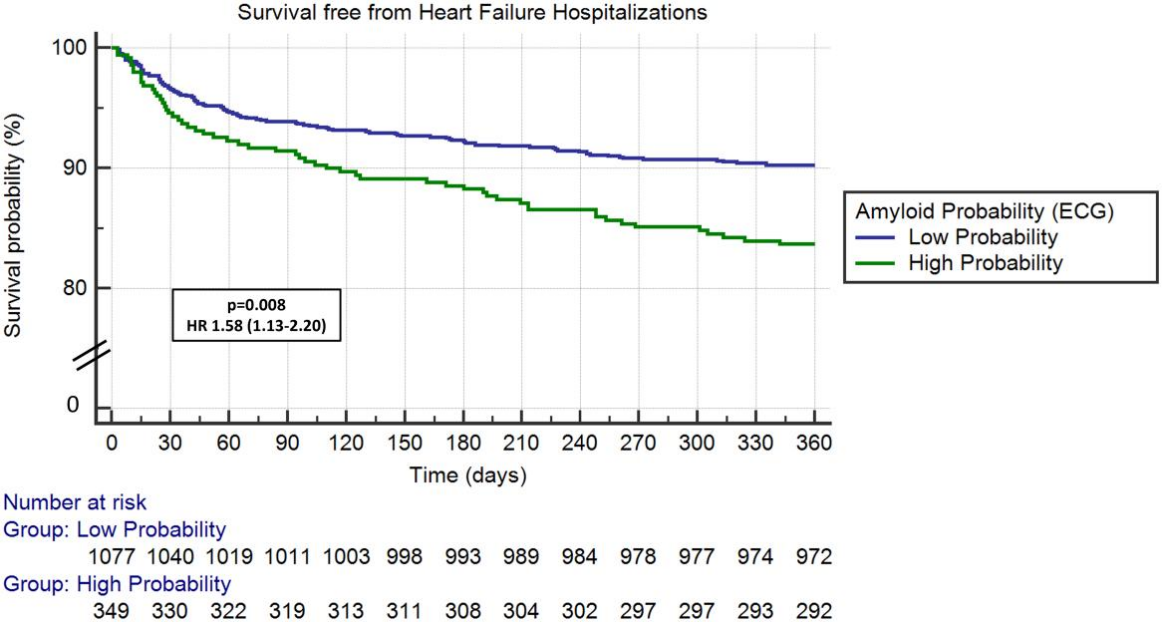
Heart failure hospitalizations in transcatheter aortic valve replacement patients with high vs. low probability for cardiac amyloidosis. ECG, electrocardiogram; HR, hazard ratio.

After adjusting for demographic characteristics and comorbidities,^11^ HR for all-cause mortality (HR 1.40, 95% CI 1.01–1.96, *P* = 0.046), MACE (HR 1.36, 95% CI 1.01–1.82, *P* = 0.041), and HF hospitalizations (HR 1.58, 95% CI 1.13–2.20, *P* = 0.008) remained significantly increased for patients in the high probability for CA group (*Table 2*). *Figure 2* demonstrates Kaplan–Meier curves for all-cause mortality, *Figure 3* for MACE and *Figure 4* for HF hospitalizations at 1-year follow-up for patients with high probability for CA group compared with those with low probability.

**Table 2:** Unadjusted and adjusted hazard ratios for clinical outcomes at 1-year follow-up for those at higher probability for cardiac amyloidosis as identified by the electrocardiogram artificial intelligence algorithm

	Unadjusted		Adjusted by propensity score	
Outcomes at 1-year follow-up	Hazard ratio (95% CI)	*P*-value	Hazard ratio (95% CI)	*P*-value
Mortality	1.51 (1.10–2.08)	0.012	1.40 (1.01–1.96)	0.046
MACE	1.48 (1.12–1.97)	0.006	1.36 (1.01–1.82)	0.041
Heart failure	1.69 (1.23–2.33)	0.001	1.58 (1.13–2.20)	0.008
TIA/stroke	1.14 (0.63–2.06)	0.667	1.07 (0.58–1.97)	0.840
Myocardial infarction	1.03 (0.33–3.18)	0.966	0.92 (0.29–2.98)	0.895

When comparison was made by quartiles of ECG AI probability for CA, patients in the highest quartile had increased incidence of mortality both in the adjusted and unadjusted analysis, with adjusted HR 1.98, 95% CI 1.23–3.18, *P* = 0.005, and unadjusted HR 2.15, 95% CI 1.36–3.40, *P* = 0.001. Patients in the highest quartile also had increased risk for MACE (HR 1.63, 95% CI 1.13–2.37, *P* = 0.010), however, did not reach significance despite trending towards it with propensity score covariate adjustment (HR 1.46, 95% CI 0.99–2.15, *P* = 0.054). Those in the highest quartile had increased risk for HF hospitalization, with adjusted HR 1.84, 95% CI 1.17–2.90, *P* = 0.008 and unadjusted HR 2.01, 95% CI 1.29–3.12, *P* = 0.002.

## Discussion

An AI algorithm that is clinically available in our institution’s EMR and previously validated as being predictive for the presence of CA^9^ has prognostic value for mortality and MACE in those it identified as at high risk for CA when applied to pre-TAVR ECGs in patients with severe AS. Patients identified as high risk for CA had increased hospitalization for HF, which is relevant in the CA population. However, there was no significant difference in stroke or MI events that occurred less frequently at follow-up. This study highlights the translational application and clinical utility of AI for the prediction of cardiovascular disease and outcomes by ECG. As such, AI applied to pre-TAVR ECGs may be useful as a screening tool to identify patients with an increased probability for CA and at higher risk of clinical events.

Cardiac amyloidosis is an infiltrative process caused by the deposition of amyloid fibrils within cardiac structures. The most common amyloid protein affecting the heart is transthyretin (TTR), which predominately affects older adults.^6^ Accumulation of amyloid fibrils in the myocardium can lead to HF and conduction abnormalities. Amyloid can also infiltrate cardiac valves, where associated endothelial damage may lead to subsequent calcification, with the aortic valve being most commonly affected.^12^ Several studies have shown a high prevalence of TTR CA in patients with severe AS undergoing TAVR. Castaño *et al*. performed PYP scans in 151 patients with severe calcific AS who underwent TAVR, showing that TTR CA was prevalent in 16%.^5^ Other investigations have suggested a prevalence of TTR CA of 13% in patients with severe AS undergoing TAVR.^6,13^

Cardiac amyloidosis in general is associated with poorer prognosis.^14^ Cardiac amyloidosis has traditionally been associated with worse clinical outcomes post-TAVR.^3,6,15^ A recent meta-analysis of 1321 patients with AS demonstrated higher mortality rates in those with CA than those with AS alone,^16^ despite a recent prospective study with small numbers suggesting to the contrary—a similar prognosis at 1-year follow-up.^17^ In this group of patients undergoing a significant procedure such as TAVR, identifying CA early is important for better risk stratification, improved surveillance post-procedure, and permitted access to now available treatments and specialized amyloid clinics for care, which may improve outcomes.^4,18^

Cardiac amyloidosis can be further evaluated with 99mTc-PYP scan that has a high negative predictive value for TTR CA, FLC assays to assess for AL CA, cardiac magnetic resonance imaging (MRI) that may show diffuse infiltration on late gadolinium-enhanced sequences, and endomyocardial biopsy.^17,19^ In particular, PYP scan and cardiac MRI have been suggested to screen for CA in all patients undergoing TAVR; however, this would be costly and cumbersome for patients who would have additional burden of testing over and above the multiple tests required in the pre-TAVR evaluation, and access may be limited. Specifically, the average cost of a nuclear PYP scan is between $3000 and $7000 in the USA without insurance.^16^ At an estimated detection rate of one in nine post-TAVR patients, the cost of detection of one TTR CA patient would be between $27 000 and $63 000. Given these challenges, testing for CA in patients with severe AS is not routinely performed, despite its high prevalence and associated worse prognosis.

Advances in AI have created revolutionary and unique opportunities to predict cardiovascular pathology from tests that may appear normal to the human eye.^20,21^ The first such development with ECG was a convolutional neural network model to detect the electrocardiographic signature and predict atrial fibrillation development from ECGs in normal sinus rhythm.^22,23^ Similarly, Grogan *et al*. from our same institution developed and validated the AI predictive algorithm for predicting CA from routine ECG that was used in the current study; this model had excellent performance for diagnostic correlation for the presence of CA with an AUC (area under the curve) of 0.91 (95% CI 0.90–0.93).^9^ The ECG predictive model for CA applied an optimal probability threshold of 0.485, with a sensitivity of 0.84 and a specificity of 0.85. The model was developed on the hypothesis that ECG changes in CA develop well ahead of overt clinical manifestation and that an increased probability of CA diagnosis can be detected by the application of AI to routine clinical ECG.^9,10^

To the best of our knowledge, this is the first study to analyse the AI-predicted risk of CA applied to ECGs of patients undergoing TAVR and its relationship with cardiovascular outcomes. The ability to identify patients with poorer prognosis and potentially higher likelihood of undiagnosed CA with an inexpensive, widely available, point-of-care test as the surface ECG has paramount practical implications, particularly for CA screening prior to TAVR. In our study, only 17 (1.1%) patients from the overall cohort (*n* = 1426) had a clinical diagnosis of CA, which is reflective of most real-world practices where patients with severe AS undergoing TAVR do not undergo dedicated comprehensive evaluation for CA.

However, the true prevalence of CA in the TAVR population is significantly higher, with prior studies utilizing comprehensive mass screening for TTR CA reporting a 13–16% prevalence.^24,25^ The diagnostic value of the AI algorithm applied to ECGs has been previously reported; this study demonstrates the prognostic value for MACE at a relatively short follow-up of 1 year. Accordingly, the AI predictive ECG algorithm may be useful clinically as a cost-effective screening tool, where it may identify a subgroup of patients that may benefit from further dedicated diagnostic evaluation for CA, especially in an era where novel treatments are available. Diagnosis of CA as may be facilitated by this AI algorithm may permit closer attention to guideline-directed medical therapy for HF was present, the commencement of targeted amyloid therapy if indicated, and allow the benefits of multidisciplinary amyloid specialized clinic care.

### Limitations

Our findings may be limited by the retrospective nature of the study, although a propensity score covariate adjustment model was applied to adjust for potential confounders. The time for the development of clinically evident CA and time to diagnosis may be prolonged because of lack of suspicion or diagnostic workup, and further prospective studies are required to assess for clinical diagnosis of CA based on high probability prediction based on the AI model. A systematic evaluation for CA, especially with PYP screening across the entire cohort, was not available to determine the true prevalence of CA. However, this reflects real-world practice and lack of feasibility in terms of mass screening with nuclear imaging, although the literature provides insight of the more common prevalence of CA in this population as noted.

## Conclusion

Artificial intelligence applied to pre-TAVR ECGs identifies a subgroup at high risk for CA who have worse clinical outcomes. Artificial intelligence may be useful to screen and identify patients at high risk who may benefit from further dedicated diagnostic evaluation for CA.

## Lead author biography


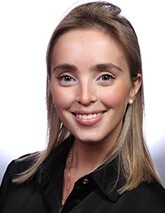
 Milagros Pereyra, MD, graduated from the Italian University Institute of Rosario (IUNIR), Argentina. She is currently a research fellow in the Department of Cardiovascular Medicine at Mayo Clinic Arizona and pursuing a career in cardiology. Her main areas of interest are cardiovascular imaging and the application of artificial intelligence in the cardiology field.

## Data Availability

The data presented in this study are available on request from the corresponding author.
